# A comprehensive mapping of outcomes following psychotherapy for adolescent depression: The perspectives of young people, their parents and therapists

**DOI:** 10.1007/s00787-020-01648-8

**Published:** 2020-10-01

**Authors:** K. Krause, N. Midgley, J. Edbrooke-Childs, M. Wolpert

**Affiliations:** 1 Research Department for Clinical, Educational and Health Psychology, University College London, Gower St, Bloomsbury,, WC1E 6BT London UK; 2 Evidence-Based Practice Unit, Anna Freud National Centre for Children and Families, 4-8 Rodney Street, London, N1 9JH UK; 3 Child Attachment and Psychological Therapies Research Unit (ChAPTRe), Anna Freud National Centre for Children and Families, 4-8 Rodney Street, London, N1 9JH UK; 4 Wellcome Trust, 215 Euston Rd, Bloomsbury, London, NW1 2BE UK

**Keywords:** Depression, Outcome, Qualitative, Adolescents, Psychotherapy, Patient perspectives

## Abstract

As mental health systems move towards person-centred care, outcome measurement in clinical research and practice should track changes that matter to young people and their families. This study mapped the types of change described by three key stakeholder groups following psychotherapy for depression, and compared the salience of these outcomes with the frequency of their measurement in recent quantitative treatment effectiveness studies for adolescent depression.

Using qualitative content analysis, this study identified and categorized outcomes across 102 semi-structured interviews that were conducted with depressed adolescents, their parents, and therapists, as part of a randomized superiority trial. Adolescents had been allocated to Cognitive-Behavioral Therapy, Short-Term Psychoanalytic Psychotherapy, or a Brief Psychosocial Intervention.

The study mapped seven high-level outcome domains and 29 outcome categories. On average, participants discussed change in four domains and six outcome categories. The most frequently discussed outcome was an improvement in mood and affect (i.e., core depressive symptoms), but close to half of the participants also described changes in family functioning, coping and resilience, academic functioning, or social functioning. Coping had specific importance for adolescents, while parents and therapists showed particular interest in academic functioning. There was some variation in the outcomes discussed beyond these core themes, across stakeholder groups and treatment arms.

Of the outcomes that were frequently discussed in stakeholder narratives, only symptomatic change has been commonly reported in recent treatment studies for adolescent depression. A shift towards considering multiple outcome domains and perspectives is needed to reflect stakeholder priorities and enable more nuanced insights into change processes.

**Electronic supplementary material:**

The online version of this article (10.1007/s00787-020-01648-8) contains supplementary material, which is available to authorized users.

Depression is a common mental health problem in adolescence and one of the leading causes of health-related disability amongst young people worldwide [1]. The lifetime prevalence of depression during adolescence is estimated at 11.0% in the United States [2], 11.4% in Sweden, and 15.5% in the Netherlands [3]. Adolescent-onset depression can negatively impact on physical health [4, 5], educational attainment, and employment [6–10] over the life course. Identifying efficacious treatments and ensuring their effective delivery in routine care are pressing priorities. Meaningful data on treatment outcome are central to this effort.

Traditionally, outcome measurement for adolescent depression has focused on change in depressive symptoms, which include low mood or loss of pleasure in daily activities, sleeping difficulties, loss of energy, changes in weight or appetite, suicidal thoughts or behavior, anger and irritability [11, 12]. A recent systematic review found that 94% of treatment efficacy and effectiveness studies for adolescent depression published between 2007 and 2017 tracked change in depressive symptoms, while less than 10% tracked change in outcome domains such as interpersonal relationships (e.g., family functioning), personal growth (e.g., self-esteem, autonomy), or quality of life. The extent to which this focus on symptom change reflects what matters most to service users and their families is unclear [12]. As health care systems strive to become more person-centred [13], outcome measurement should reflect what matters most to young people and their families [14, 15].

Qualitative research has an important role in providing a deeper understanding of therapy outcome from the perspective of key stakeholder groups [16], which include young people, parents,^1^ and therapists. Existing studies exploring their notions of ‘good outcome’ have drawn attention to a broader range of themes, as well as divergent priorities between groups. Adolescents have tended to emphasize changes in their ability to understand and cope with feelings and thoughts; greater connectedness with others; a stronger sense of self; and increased hope [17–21]. Parents have been found to value outcomes related to managing youth behavior; and therapists have been seen to focus on intermediate outcomes linked to their training and approach [22, 23]. Disagreement between youth, parents, and therapists on the most important treatment goals has been frequently observed [21, 23–26]. This is in line with the tripartite model of outcome suggested by Strupp and Hadley [27], whereby different stakeholders judge outcomes from different vantage points. Yet, all three groups have expressed a preference for outcome measurement that is meaningful, can be adapted to the needs and complexities of individual cases, captures change holistically, and represents more than a box-ticking exercise [28–33].

Most existing qualitative studies have employed heterogeneous samples, including children and adolescents with a range of presenting problems, without disaggregating findings for specific subgroups. It remains unclear what outcomes stakeholders value specifically for adolescent depression. In addition, most studies have assessed desired outcomes and disagreement between stakeholders at the start of treatment. Notions of outcome may, however, evolve over the course of therapy, and divergent priorities may gradually converge [24]. Indeed, the post-treatment perspective has been described as the most informative for investigating outcome perceptions [34].

To date, no qualitative study has systematically analyzed post-treatment outcome perceptions specifically in relation to depression amongst young people, parents, and therapists. In addition, no study has systematically assessed the extent to which outcomes valued by these key stakeholder groups are measured and reported in quantitative treatment outcome studies for adolescent depression. This study aims to address these gaps by providing a systematic mapping of outcomes described by adolescents, parents, and therapists following treatment in three different arms of a psychotherapy trial for adolescent depression. We mapped outcomes using qualitative content analysis, with a taxonomy of treatment outcome serving as an initial coding frame. An earlier version of the same taxonomy was used in a systematic review that mapped outcomes measured and reported in quantitative treatment outcome studies [35], thus enabling a comparison of outcome themes and their salience.

## Method

### Setting

This study is a post-hoc analysis of interview data collected through the qualitative IMPACT-My Experience (IMPACT-ME) study [36]. IMPACT-ME was nested within the IMPACT trial, a pragmatic effectiveness superiority trial of psychotherapeutic treatments for adolescent depression [37]. The IMPACT trial randomized 467 clinically depressed adolescents across 15 specialist child and adolescent mental health services in England to one of three psychological therapies. IMPACT-ME aimed to complement quantitative outcome assessment using standardized measures with the qualitative longitudinal exploration of change through semi-structured interviews, at three time points (i.e., at the start and end of treatment, and at one-year follow-up).

### Interventions

The IMPACT trial had three treatment arms: a Brief Psychosocial Intervention (BPI), Cognitive Behavioral Therapy (CBT), and Short-Term Psychoanalytic Psychotherapy (STPP) [37]. BPI involved psychosocial management over 20 weeks, with up to 12 sessions for adolescents, with flexible involvement of family members. The focus was on psychoeducation, behavioral activation, problem solving, risk management and physical and mental hygiene [38, 39]. CBT involved up to 20 individual sessions over 30 weeks, plus up to 4 family or parental sessions, and focused on identifying and challenging negative automatic thoughts and their linkage with behavior, and on developing more adaptive cognitive and behavioral techniques [38, 40]. STPP comprised up to 28 sessions over 30 weeks with the option of parents accessing additional sessions with a parent worker. Using a psychodynamic approach, therapists guided young people in expressing and interpreting difficult feelings and experiences through a non-judgmental process [38, 41–43].

### Participants

Adolescents aged 11–17 years with a current DSM-IV diagnosis of unipolar Major Depressive Disorder with moderate to severe functional impairment were eligible for the IMPACT trial [37]. Exclusion criteria included generalized learning difficulties or a pervasive developmental disorder; a substance use disorder; a primary diagnosis of bipolar disorder, schizophrenia, or eating disorder; pregnancy; use of medication that could interfere with pharmacotherapy for depression; and having completed one of the study treatments in the past [38].

Trial participants at the five London-based centers could join the qualitative IMPACT-ME study. Semi-structured interviews were conducted individually with adolescents, their parents, and (if the young people consented) their IMPACT therapists. The present analysis focused on the post-treatment interviews (timepoint 2), and only considered cases for whom all three members of a triad had been interviewed. This was true for 40 cases, of which five adolescents had dropped out of treatment within the first three sessions, and one had been referred to inpatient care. These six cases were excluded from analysis, to focus on outpatient experiences of a minimum length. The final analytical sample comprised 102 interviews across 34 triads. At the time of the post-treatment interview, adolescents were aged 16.2 years on average (*SD* = 1.5; range = 12–19), and 21 (62%) were female. Nine had been treated in the BPI arm, nine in the CBT arm, and 16 in the STPP arm.

### Data collection method

IMPACT-ME encouraged participants to provide in-depth accounts of their experiences in their own words [16]. Semi-structured interviews were conducted using the Experience of Therapy Interview guide [ETI; 44], which was tailored to each participant group in wording, but similar in content. Participants were asked about how things were for them now compared to when treatment started, any changes they had observed since the start of treatment and how they understood those changes, their experience of therapy, including any helpful or unhelpful aspects, and any significant moments or turning points. Interviews each lasted between 30 and 60 min. They were conducted by research psychologists, recorded, and transcribed verbatim for analysis.

### Data analysis

This study used qualitative content analysis to map the outcomes discussed by participants. Contrary to classic content analysis, qualitative content analysis moves beyond the counting of words or expressions to the examination of patterns of explicit or inferred meaning in participant narratives, their subjective interpretation, and systematic classification [45, 46]. Like thematic analysis [47], qualitative content analysis involves the coding of data into categories but ends with quantifying their occurrence rather than aggregating them into higher-level themes [48]. Qualitative content analysis is well suited for systematically condensing a phenomenon into a conceptual framework, especially with large data volumes [45]. It can be used inductively or deductively [49].

We chose a deductive approach, by applying a taxonomy of treatment outcome as an a-priori coding frame. This approach was chosen to enhance the comparability and transparency of the resulting outcome mapping, and to “canvass the full range” of potentially relevant outcome categories [50]. The taxonomy was derived as part of a separate study, which aimed to identify, critically appraise, and synthesize existing outcome taxonomies relevant to child and adolescent mental health. That study identified three relevant conceptual models of outcome [51–53] and three-goal taxonomies [20, 54, 55]. Outcome categories included in these primary frameworks were extracted, tabulated [50], and appraised for their relevance to a person-centred examination of outcomes for adolescent depression. If considered relevant, these categories were then synthesizd into a new, more comprehensive taxonomy. Details about this taxonomy and its development can be obtained from the authors upon request.

We used this initial taxonomy to organize descriptions of change in participant narratives into outcome categories. We focused on the semantic content of the data but moved beyond the coding of specific words to the interpretation and categorization of passages within their narrative context, by considering implicit as well as surface meanings. The initial taxonomy was iteratively revised to reflect new themes emerging from the data, by creating, modifying, merging, or removing categories until saturation was reached—thus adding an inductive element to the coding process. The final coding frame (see Online Resource 1) consists of seven high-level outcome domains (*symptoms*, *self-management*, *functioning*, *personal growth*, *relationships*, *youth wellbeing*, and *parental support and wellbeing*), and 29 specific outcome categories (see Table 1).

**Table 1:** Percentage of participants reporting each outcome category versus measurement in quantitative outcome studies

Outcome domain and subdomain	Literature^a^		Participant narratives													
	(*k* = 92)		Full sample (*n* = 102)		Adolescents (*n* = 34)		Parents (*n* = 34)		Therapist (*n* = 34)		CBT (*n* = 27)		STPP (*n* = 48)		BPI (*n* = 27)	
	*k*	%	*n*	%	*n*	%	*n*	%	*n*	%	*n*	%	*n*	%	*n*	%
Symptoms	86	93%	81	79%	28	82%	26	77%	27	79%	24	90%	35	73%	22	82%
Mood & affect	86	93%	66	65%	22	65%	21	62%	23	68%	22	82%	27	56%	17	63%
Anger and aggression	4	4%	17	17%	8	24%	8	24%	1	3%	4	15%	9	19%	4	15%
Eating and weight	1	1%	13	13%	3	9%	6	18%	4	12%	3	11%	4	8%	6	22%
Sleeping and energy	2	2%	20	20%	5	15%	8	24%	7	21%	6	22%	7	15%	7	26%
Self-harm	1	1%	12	12%	4	12%	3	9%	5	15%	3	11%	6	13%	3	11%
Suicidality	15	16%	14	14%	8	24%	2	6%	4	12%	1	4%	8	17%	5	19%
Anxiety	7	8	12	12%	4	12%	3	9%	5	15%	4	15%	1	2%	7	26%
Other comorbid issues	9	10%	4	4%	2	6%	2	6%	—	—	—	—	2	4%	2	7%
Self-management	14	15%	62	61%	24	71%	20	59%	18	53%	20	74%	25	52%	17	63%
Behavioral activation	4	4%	20	20%	6	18%	6	18%	8	24%	7	26%	4	8%	9	33%
Coping and resilience	2	2%	51	50%	22	65%	17	50%	12	35%	16	59%	23	48%	12	44%
Cognition and behavior	9	10%	19	19%	8	24%	6	18%	5	15%	12	44%	4	8%	3	11%
Functioning	51	55%	67	66%	19	56%	26	77%	22	65%	24	89%	26	54%	17	63%
Global functioning	48	52%	9	9%	2	6%	3	9%	4	12%	—	—	4	8%	5	19%
Executive functioning	2	2%	20	20%	8	24%	8	24%	4	12%	9	33%	9	19%	2	7%
Academic and vocational functioning	0	0%	46	45%	10	29%	18	53%	18	53%	15	56%	19	40%	12	44%
Social functioning	3	3%	36	35%	12	35%	14	41%	10	29%	13	48%	13	27%	10	37%
Personal growth	7	8%	70	69%	23	68%	24	71%	23	68%	18	67%	36	75%	16	59%
Assertiveness	1	1%	13	13%	4	12%	3	9%	6	18%	1	4%	8	17%	4	15%
Autonomy and responsibility	1	1%	16	16%	3	9%	10	29%	3	9%	3	11%	9	19%	4	15%
Identity	6	7%	14	14%	1	3%	2	6%	11	32%	3	11%	8	17%	3	11%
Processing past and present	—	—	18	18%	6	18%	5	15%	7	21%	7	26%	6	13%	5	19%
Confidence and self-esteem	1	1%	34	33%	10	29%	13	38%	11	32%	7	26%	16	33%	11	41%
Feeling seen and seeing differently	—	—	29	28%	13	38%	10	29%	6	18%	8	30%	16	33%	5	19%
Relationships	4	4%	63	62%	21	62%	22	65%	20	59%	20	74%	26	54%	17	63%
Ability to communicate feelings and thoughts	—	—	13	13%	4	12%	8	24%	1	3%	3	11%	6	13%	4	15%
Family functioning and relationships	4	4%	50	49%	16	47%	17	50%	17	50%	18	67%	18	38%	14	52%
Friendships	1	1%	28	27%	11	32%	9	27%	8	24%	10	37%	11	23%	7	26%
Other peer relationships	1	1%	9	9%	5	15%	—	—	4	12%	—	—	2	4%	7	26%
Wellbeing	7	8%	38	37%	9	27%	18	53%	11	32%	13	48%	15	31%	10	37%
Peace of mind	—	—	14	14%	2	6%	9	27%	3	9%	6	22%	6	13%	2	7%
Hope and optimism	—	—	12	12%	5	15%	4	12%	3	9%	6	22%	4	8%	2	7%
Future orientation	—	—	19	19%	4	12%	9	27%	6	18%	4	15%	9	19%	6	22%
Parental support and wellbeing	3	3%	24	24%	3	9%	16	47%	5	15%	2	7%	12	25%	10	37%
Parental support	—	—	7	7%	—	—	6	18%	1	3%	—	—	6	13%	1	4%
Parental wellbeing	2	2%	22	22%	3	9%	14	41%	5	15%	2	7%	11	23%	9	33%

^a^“Literature” refers to the 92 quantitative treatment efficacy and effectiveness studies for adolescent depression reviewed by KK, JEC and MW as part of a previously published systematic review [35]

The frequencies reported relate to the number of participants describing an outcome in their interview, relative to the full number of participants in the reference group (i.e., the full sample; the relevant participant group; or participants in the relevant treatment arm). We did not consider the frequency at which outcomes were discussed *within* individual interviews. We compared the frequency at which outcomes were discussed by participants with the frequency at which the same outcomes were reported in 92 quantitative treatment efficacy and effectiveness studies for adolescent depression that were published between 2007 and 2017, and reviewed by three of the authors as part of a separate systematic review [35]. The review applied an earlier version of the same outcome taxonomy used in the present study, to guide outcome categorization.

The authors conducted this study according to a pragmatist research paradigm [56–60], influenced by the association of two authors (KK, JEC) with a research unit for evidence-based child mental health, and a focus amongst all authors on generating knowledge that can promote high-quality and person-centred care. Pragmatism refutes notions whereby qualitative and quantitative methodologies, commonly associated with constructivist and positivist paradigms, respectively, are incompatible. It marks an epistemological and methodological “middle position” [57], and advocates for the use of mixed methods while acknowledging that the researcher’s values inevitably influence the interpretation of results [61]. With qualitative content analysis, we used an analytic approach rooted in a positivist paradigm. At the same time, we consider outcome narratives to be the product of co-creation by participants and researchers, and to be socially constructed. We further recognize that although three authors (KK, JEC, MW) had no direct involvement in conducting the semi-structured interviews, their training and expectations likely influenced data coding and interpretation [62, 63]. For example, the first author (KK) approached this research with an interest in identifying a range of possibly relevant outcomes, which may have led to a focus on distinguishing rather than aggregating related outcome concepts in the final coding frame.

### Ethical considerations and approval

The original study protocol for the IMPACT trial and the IMPACT-ME study were approved by Cambridgeshire 2 Research Ethics Committee, Addenbrookes Hospital Cambridge, UK (REC Ref: 09/H0308/137), and were performed in accordance with the ethical standards laid down in the 1964 Declaration of Helsinki and its later amendments. All participants above the age of 16 provided informed written consent. Parental consent and youth assent were obtained for younger adolescents. To ensure confidentiality, the interview data were anonymized, any identifying details removed, and adolescents’ names replaced with pseudonyms.

### Findings

**Fig. 1:**
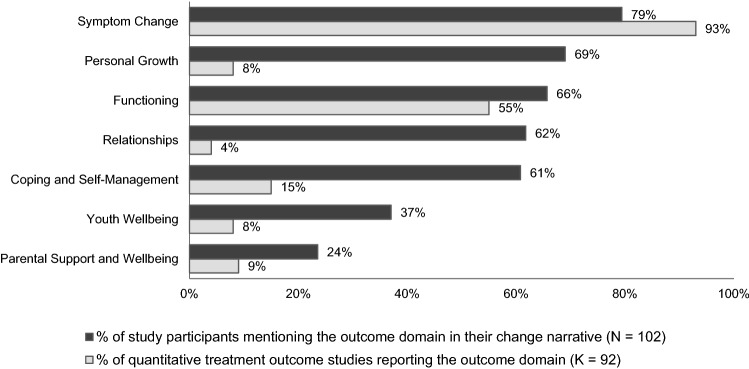
Salience of Outcome Domains in Post-Treatment Narratives Versus Quantitative Treatment Outcome Studies

Outcome narratives were distinctly multidimensional. On average, each participant discussed outcomes in relation to four domains and six more specific outcome categories. Changes in the domains of *symptoms*, *functioning*, *relationships*, *self-management*, and *personal growth* were each discussed by more than 60% of participants, and changes in *youth wellbeing* and *parental wellbeing and support* were discussed by a third, and a quarter of participants, respectively (see Fig. 1). The five most frequently discussed specific outcome categories were improvements in *mood and affect*, *coping skills and resilience*, *family functioning and relationships*, *academic and vocational functioning*, and *social functioning*. Illustrative quotes for all outcome domains and categories are provided in Online Resource 1.

Improvements in *mood and affect* were the most frequently discussed outcome across participant groups. Participants described adolescents feeling less low, withdrawn, or prone to mood swings; and more cheerful. Some described adolescents returning to “being the person they used to be” as symptoms lifted, or that they felt or appeared like a completely different person, compared with their former depressed self. Others described that low mood and negative affect were still present, but more fleeting, and less overwhelming, which was often linked to young people learning to cope more effectively.

Improvements in *coping skills and resilience* were discussed just as often as *mood and affect* by adolescents, but less often by parents and therapists. Participants described that adolescents had learned techniques (e.g., breathing or counting exercises; keeping of thought diaries) or developed personal strategies to cope with feelings and thoughts (e.g., allowing themselves to cry when feeling sad, rather than letting feelings build up), which helped combat symptoms and strengthen their self-efficacy, sense of control, and resilience. Another aspect of coping was gaining a better understanding of feelings and thoughts, and becoming more able to anticipate and manage challenging situations:

It did wake me up to how my-, sort of how it all works and like how my brain works […] the fact that if you can understand something you can fix something that’s my motto. So, if I can understand like in a computer game if I can understand why it’s not working, I can fix the problem. (Dylan, 16 years, STPP).

Improvements in *family functioning* were a prominent outcome theme across all three participant groups. Narratives were multifaceted: Some adolescents adjusted their roles within the family system by learning to impose boundaries between their needs and those of family members; some families grew closer by communicating more openly; some reported a decrease in conflict as adolescents learned to cope more effectively and family members grew more understanding; and others felt that therapy had taught them to tolerate a ‘healthy’ amount of conflict. Some adolescents were able to clarify a fraught relationship with a family member by processing resentment and learning to interact differently:

I know it sounds weird, but I can hold a good conversation with [stepfather] now […] And I kind of realize now that it wasn’t his fault and it’s never really been an issue with him just the fact that out of all the things that were going wrong, he was the one thing which was…I could blame everything on. And it’s realizing that and it’s knowing that it’s not his fault that have made it like seem easy to talk to him now and I have a really good relationship with him now and it makes everything so much easier. (Ella, 15 years, BPI).

The outcome category of *academic and vocational functioning* involved changes in attendance, commitment, and performance at school or college. Improved attendance involved adolescents missing fewer hours or days of class or returning after a sustained period of leave. Frequently, participants also described young people being better able to motivate themselves and commit to their schoolwork, which was often associated with superior grades and exam results.

Within the outcome category of *social functioning,* participants described changes in adolescents’ ability and willingness to engage with others, touching upon social skills (i.e., being better able to start and maintain conversations and relate to others; becoming more approachable; and being more mindful of other people’s feelings), as well as sociability (i.e., becoming more outgoing and talkative, more present within friendship groups, and more socially connected).

Linking up with friends, I mean this was something that we worked on quite a lot: Could she bear to actually link up with people that she may not know that well just for the sake of having somebody to go in the lunch queue with. (Therapist of Jenny, 17 years, STPP).

### Comparing outcome salience in stakeholder narratives and quantitative treatment outcome studies

**Fig. 2:**
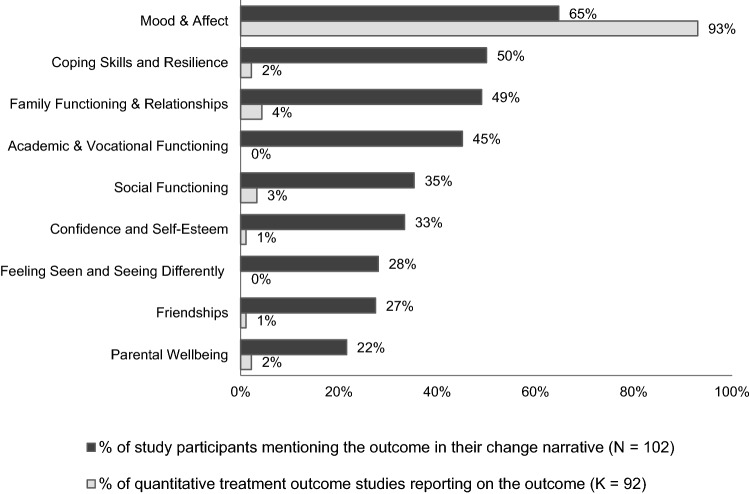
Salience of Outcome Categories in Post-Treatment Narratives Versus Quantitative Treatment Outcome  Studies

Figures 1 and 2 display how the frequency with which the seven outcome domains and the nine most frequently discussed outcome categories were discussed by study participants, compared with the frequency of their assessment in recent treatment efficacy and effectiveness studies (using standardized outcome measures) [35]. The high salience of the symptom domain, and of changes in *mood and affect* more specifically, matched the frequent reporting of symptom change in over 90% of the reviewed studies. Apart from *mood and affect*, none of the outcome categories frequently discussed in participant narratives were reported by more than 5% of the reviewed treatment studies (see Fig. 2).

Although *functioning* was also frequently assessed and reported, this was usually done via a single-item measure, such as the Clinical Global Impression (CGI) scale [64] or the Children’s Global Assessment Scale (CGAS) [65], which gauge a clinician’s overall impression of a young person’s functioning. While the CGAS encourages clinicians to consider social and academic functioning when assigning a score, it does not allow for the separate reporting of these outcomes [66]. Outcomes related to *social functioning* were *explicitly* reported in only 3% of the reviewed studies, and outcomes related to *academic and vocational functioning* in none.

### Comparing outcome salience between participant groups

Change in *mood and affect* was discussed by close to two thirds of participants across all stakeholder groups. Family functioning and relationships were discussed by around half. Improved *coping and resilience* were the second most frequently discussed outcome amongst adolescents—at par with changes in *mood and affect* (64%)—but discussed by only half of the parents and one third of therapists. Another outcome discussed considerably more often by adolescents than parents or therapists was *suicidality* (24%, versus 6% and 12%, respectively).

Over a third of adolescents further discussed experiences of *feeling seen or seeing differently* as a result of accessing therapy. For some, therapy offered the first experience of feeling truly heard and understood by another person, and of being worthy of their undivided attention.

Back then I felt like nobody cared about me and I do not I think it made me feel good within myself because it was just it’s kinda what I needed like to feel like someone … (breathes out) does care and that like they are there for me. (Natalie, 15 years, STPP).

Others described that working with the therapist opened up new perspectives. Given the transformational nature of these experience in young people’s descriptions, they might be considered as outcomes in their own right, rather than merely procedural aspects of therapy. Adolescents discussed these benefits slightly more often than parents (38% versus 29%), and more than twice as often as therapists (18%).

*Academic and vocational functioning* was the second most frequently discussed outcome theme amongst parents and therapists (53%) but discussed by less than a third of adolescents. Other outcomes discussed considerably more frequently by parents than by adolescents or therapists related to young people’s *autonomy and responsibility, ability to communicate feelings and thoughts, peace of mind*, and *future orientations* (i.e., young people’s ability to make plans and have goals for the future); as well as their own parental wellbeing and ability to provide support. Other outcomes discussed more frequently by parents related to adolescents’ *eating and weight*, *sleeping and energy*, and *social functioning*. Therapists were considerably more likely than adolescents or parents to discuss changes in young people’s *identity* (32%, compared with 3% and 6%, respectively), in terms of finding out who they are and how to be themselves around other people; and developing a more realistic self-image. In turn, close to a quarter of adolescents and parents discussed changes related to *anger and aggression*, while these were discussed by only one therapist. Similarly, the share of adolescents and parents discussing changes in *executive functioning* was twice higher than amongst therapists.

### Comparing salience between treatment arms

*Mood and affect* was the most frequently discussed outcome across all three treatment arms, but discussed most often in CBT (82%), and least often in STPP (56%). Similarly, *family functioning and relationships*, *coping and resilience*, and *academic and vocational functioning* were amongst the five most-discussed outcomes in all three arms. In CBT, the fifth most-discussed outcome was *social functioning*, while in BPI and STPP it was *self-confidence and self-esteem* (in STPP the fifth rank was shared with *feeling seen and seeing differently*).

Beyond changes in *mood and affect*, outcomes discussed considerably more frequently in the CBT arm than in BPI or STPP included *managing cognition and behavior* and *coping skills and resilience* within the domain of *self-management,* outcomes in the domains of *functioning* (with the exception of *global functioning*), *family functioning, friendships*, *peace of mind*, and *hope and optimism.* Outcomes discussed more frequently in the STPP arm than in the other two arms included *assertiveness, autonomy, identity,* and *feeling seen and seeing differently* within the *personal growth* domain; and *parental support.* Outcomes discussed more often in the BPI arm than in the two other arms included changes in *anxiety, global functioning, peer relationships*, *parental wellbeing*, *eating and weight*, *sleeping and energy*, and *behavioral activation* (see frequencies in Table 1).

## Discussion

This was the first qualitative study to comprehensively map outcomes discussed by triads of adolescents, parents, and therapists following psychotherapy for adolescent depression. It is also the first study to systematically compare the frequency at which these outcomes were discussed by participants, with the frequency of their measurement in the recent quantitative treatment outcome literature for adolescent depression; and to examine differences in outcome perceptions across participant groups and treatment arms. This study identified seven higher-level outcome domains and 29 more specific outcome themes.

Adolescents, parents, and therapists tended to reflect on change holistically, across the high-level domains of *symptoms, self-management, functioning, relationships, personal growth*, * youth wellbeing,* and *parental wellbeing and support*. A number of specific outcome categories were frequently discussed across groups and treatment arms: changes in *mood and affect* were the single most-discussed outcome, although improved *coping and resilience* was discussed just as often by adolescents; and changes in *family functioning*, and *academic and vocational functioning* were discussed by close to half of participants. Of these outcomes, only the category of *mood and affect* (i.e., core depressive symptoms) has been consistently reported in recent quantitative treatment outcome studies. In turn, family functioning was assessed in only four out of 92 reviewed studies, *coping and resilience* in two studies, and academic and vocational functioning in none [35].

Adolescents were frequently concerned with the change in their symptoms, as were their parents and therapists. This is in line with two previous studies that examined treatment goals defined by youth with mixed presenting problems in routine care [21] and school-based counselling [20]. These studies also dentified improvements in *mood and affect* as a common goal theme. Changes in affect also constituted a salient theme in interviews conducted with six Chilean adolescents following therapy for depression [17]. However, symptom change was not identified as a salient theme by a qualitative study examining notions of good outcome amongst Norwegian adolescent service users, who instead emphasized autonomy, identity, and hope. These are common themes in the adult recovery literature [18, 67], but were not frequently discussed by participants in the present study.

Our findings align with previous research in highlighting the importance of coping and resilience [17–21]; improved family functioning and relationships [17, 24, 26, 68]; social functioning and connectedness [18, 19, 67]. In addition,  a growing body of qualitative research examining procedural aspects of therapy and facilitators of good outcome is emphasizing the importance of young people feeling heard, listened to, and able to open up without feeling judged [69–74]. In this study, experiences of being worthy of another person’s attention, of feeling listened to, or of discovering new perspectives on life were described as so transformative by more than a third of adolescents, that they might be considered outcomes in their own right rather than mere facilitators of change.

We observed a greater focus on functioning amongst parents and therapists than amongst adolescents (especially in relation to academic functioning); a tendency amongst parents to discuss youth behaviors at home (e.g., sleeping and eating), and a focus on young people’s autonomy and future orientations, which aligns with existing research about parental outcome priorities [23, 24, 75]. However, previous studies focusing on youth with mixed ages and presenting problems also reported a parental emphasis on behavior management and obedience [21, 26], which was not a common theme in this study. This may reflect our focus on adolescent depression, where oppositional behavior may not constitute one of the most pressing concerns. Therapists frequently discussed changes in identity and self-confidence, which may reflect that 16 of the 34 cases received short-term psychoanalytic psychotherapy, where changes related to the sense of self are of particular concern [23, 76].

### Implications for clinical research and practice

Our findings underscore calls to review the convention of judging treatment efficacy in relation to a single primary outcome measure, which has generally been symptom-focused and clinician-reported [12, 35, 77]. Although symptom change was the most frequently discussed outcome in this study, narratives were multifaceted and touched upon additional domains, such as functioning and family relationships, where symptom scores have been shown to be an imperfect proximal indicator of change [78–80]. Adolescents, parents, and therapists provided complementary accounts of the outcome, in line with an assertion by Weisz and colleagues [81], whereby “youth therapy outcome is always, to some extent, in the eye of the beholder, and […] different informants observe different samples of a youth’s behavior, in different contexts, and bring different perspectives to what they observe” (p. 95). Exploring who observes what type of change, and under which conditions, is essential for generating nuanced understandings of treatment efficacy [77].

So-called Core Outcome Sets (COS) recommend a battery of outcomes to be measured in all trials for a given disorder, or by all those providing relevant care, as a *minimum,* to strengthen and harmonize outcome reporting [82]. COS move away from a single primary outcome measure, to reporting a set of agreed outcomes that are considered meaningful by key stakeholders. A COS for clinical trials relating to adolescent depression is currently under development at the Hospital for Sick Children in Toronto, Canada [83]. A COS for children and young people treated for anxiety and depression in routine care settings has recently been devised under the lead of the International Consortium for Health Outcomes Measurement (ICHOM) [84]. Our findings can inform such efforts by identifying candidate outcomes for inclusion in a COS. They also demonstrate the importance of considering a broad range of outcomes and stakeholder perspectives, as well as intermediate outcomes relating to specific treatment mechanisms when designing outcome standards [52, 85, 86].

Although this study has identified several possible core outcomes, there is considerable variety in outcome perspectives beyond this core. In clinical practice, services must balance a desire for tailored assessment amongst adolescent, parents, and therapists, with the burden of administering complex and lengthy questionnaire batteries. One way forward may be to consult different informants on different outcomes, to reflect specific concerns and insights (e.g., consulting parents on adolescents’ sleep hygiene, and adolescents on perceived coping skills). Another important tool for tailoring measurement beyond a core set of outcomes is the use of idiographic outcome measures that track progress in relation to individually defined target problems or treatment goals [87–89].

A third possible avenue for capturing change across different outcome domains may be through self-reported measures of functioning or Quality of Life (QoL), which track the extent to which symptoms interfere with daily functioning within the family, at school, and at home, thus covering several outcome domains at once [90]. Future research is needed to determine whether such measures are seen by young people, parents, and clinicians to produce a sufficiently holistic picture. In addition, such research should explore whether generic measures of QoL are preferred over measures of disorder-specific impairment, and whether existing scales are sufficiently sensitive to change [78].

Next to a need for broader and more personalized outcome measurement, qualitative research provides an important avenue for future inquiries about treatment outcome in adolescent depression [16]. It can complement and contextualize quantitative outcome data by providing a more holistic picture of the changes achieved, and by informing judgements about whether a young person has genuinely improved with the help of therapy [22, 36, 91]. Mixed-method inquiries about notions of ‘good outcome’ have been conducted with depressed adults [92, 93], as well as in relation to adolescent drop-out from psychotherapy using data from the IMPACT trial and IMPACT-Me study [94]. Similar mixed-methods research is needed in relation to treatment outcome in adolescent depression.

### Limitations and area for future research

This study focused on producing a comprehensive taxonomic mapping of outcomes discussed by young people, parents, and therapists following psychotherapy. We considered a maximum number of eligible interviews from the IMPACT-ME study and used an analytic technique suitable for the systematic analysis of large data volumes. This prioritization of breadth over depth came at the expense of a “thicker” inquiry into how individuals construct and understand outcome; and into more nuanced differences between stakeholder groups or treatment modalities. Future research should follow such lines of inquiry using smaller samples and more inductive and interpretative analytic approaches, with a view to advancing theory around therapeutic change. For example, Bergmans and colleagues [19] used grounded theory [95] to identify key elements of recovery from the perspective of young adults with recurrent suicidal behavior, exploring not only types of change, but also their sequencing, and the turning points that marked the transition from one phase of recovery to another. Dhanak and colleagues have used Interpretative Phenomenological Analysis (IPA) [96] to explore experiences of BPI in a small sample of five IMPACT participants to identify mechanisms contributing to good outcome [73]. IPA could be used in future studies to closely examine and interpret individual accounts of the outcome, and position them within a wider social, cultural, or theoretical, context [97].

We did not examine outcome salience at an individual level, that is, with respect to the frequency at which individuals discussed certain outcomes relative to others. We placed the focus on overarching group-level findings, which brings obvious limitations in terms of what can be grasped at the level of subjective experiences. Future research could investigate differential salience of outcomes at an individual level, and attempt to identify priority profiles using dedicated techniques such as ideal-type analysis [98], or Q-methodology [99, 100].

As qualitative research relies on narratives, it risks favoring the most articulate and confident voices. Although we coded even short descriptions of change, our analysis may not fully grasp the experiences of youth less able or willing to provide detailed and articulate verbal accounts of their experience. In addition, the study sample was limited to participants from the Greater London area, who may not be representative of adolescents, parents, or therapists in other regions of the United Kingdom, or indeed, from elsewhere in the world.

It was not always possible to disentangle outcomes enabled by therapy from changes caused by external factors. Any change mentioned by participants was coded, based on the understanding that the *type* of change appeared meaningful regardless of whether therapy had succeeded in bringing it about. The change narratives provided by adolescents and parents may further have been influenced by the values, terminology, or priorities conveyed by therapists during treatment [34]. Comparing the frequency at which outcomes were discussed between treatment arms aimed to make this more transparent. The observed focus on symptom change may also have been influenced by adolescents and parents completing standardized measures of depressive and comorbid symptoms, psychosocial impairment, and health-related quality of life as part of the IMPACT trial protocol [38].

Ideally, the credibility of this analysis would have been strengthened by having a co-analyst replicate the coding independently, and by validating the emerging categories with the original study participants [62]. Co-analysis was made difficult by the large data volume and the iterative process of devising the final coding frame. This post-hoc analysis was conducted following the end of the IMPACT-ME study, and the authors had no ethical clearance to re-contact participants for the purpose of validation.

## Conclusions

This study highlights that adolescents, parents, and therapists consider a range of outcomes when reflecting on change observed over the course of therapy for depression. While change in *mood and affect* (i.e., a reduction in core depressive symptoms) was the most frequently discussed outcome theme, change was also frequently discussed in relation to *coping and resilience*, *family functioning*, *academic and vocational functioning*, and *social functioning.* These outcomes were salient across stakeholder groups and treatment arms. Outcomes discussed beyond these core categories revealed differences in perspectives and priorities between stakeholders, and treatment arms. Only symptomatic change has been commonly measured and reported in recent treatment efficacy and effectiveness studies for adolescent depression. Clinical research and practice would benefit from establishing new standards for outcome measurement that consider multiple domains and perspectives, reflecting stakeholder priorities. Such standards should include an element of personalized assessment to ensure that the outcomes tracked are of personal relevance to service users, especially in routine care settings. Finally, qualitative research has an essential role in moving the field towards a more nuanced understanding of change achieved through psychotherapy, and in complementing and contextualizing quantitative outcome measurement.

## Availability of data and material

Not applicable.

## Electronic supplementary material

Below is the link to the electronic supplementary material.

Supplementary file1 (DOCX 61 kb)

## Data Availability

Not applicable.

## Footnotes

1 For the sake of brevity, the term “parent” will henceforth be used to refer to parents, as well as other primary caregivers.
