# Supplementary material for: A comprehensive mapping of outcomes following psychotherapy for adolescent depression: The perspectives of young people, their parents and therapists
Source: Eur Child Adolesc Psychiatry. 2020 Oct 1;30(11):1779–91. doi: 10.1007/s00787-020-01648-8 (PMC8558204; doi:10.1007/s00787-020-01648-8)
Supplement: Supplementary file 1 — Supplementary file1 (DOCX 61 kb) [file 787_2020_1648_MOESM1_ESM.docx]

RUNNING HEAD: MAPPING OF PSYCHOTHERAPY OUTCOMES

ONLINE SUPPLEMENT

**A Comprehensive Mapping of Outcomes Following Psychotherapy for Adolescent Depression: The Perspectives of Young People, their Parents and Therapists**

Karolin Krause, PhD^1,2^, Nick Midgley, PhD^1,3^, Julian Edbrooke-Childs, PhD^1,2^, Miranda Wolpert, DClinPsych^1,4^

1. Research Department for Clinical, Educational and Health Psychology, University College London, Gower St, Bloomsbury, London WC1E 6BT, UK
2. Evidence-Based Practice Unit, Anna Freud National Centre for Children and Families, 4-8 Rodney Street, London, N1 9JH, UK
3. Child Attachment and Psychological Therapies Research Unit (ChAPTRe), Anna Freud National Centre for Children and Families, 4-8 Rodney Street, London, N1 9JH, UK
4. Wellcome Trust, 215 Euston Rd, Bloomsbury, London NW1 2BE, UK

**Corresponding Author**: Karolin Krause, Evidence-Based Practice Unit, University College London and Anna Freud National Centre for Children and Families, the Kantor Centre of Excellence, 4-8 Rodney Street, London N1 9JH, United Kingdom, [karolin.krause.16@ucl.ac.uk](mailto:karolin.krause.16@ucl.ac.uk).

# Final Coding Frame for the Qualitative Content Analysis

**Table S1.** Code Descriptions, Definitions, and Illustrative Quotes

| Outcome domain | Outcome category | Description | Example quote | | |
| --- | --- | --- | --- | --- | --- |
| Symptom change | Mood and affect | YP are less low and depressed, happier, and more cheerful, less prone to mood swings, less withdrawn.  YP return to be the person they used to be or appear to be a new person.  Low mood and negative affect are more fleeting, less overwhelming (often linked to YP coping) | “She seemed to be happy, you seemed to be able to have a joke with her and a laugh with her and ... she was just generally happier in herself.” (Mother of Madeline, 15 yrs, CBT)  “If I hadn’t gone there, I think I’d be a completely different person, like I’d still be really low now.” (Natalie, 15 yrs, STPP )  “A lot is different with (name). She’s sort of made a full recovery if you like.” (Therapist of Louise, 17 yrs, CBT) | | |
|  | Anger and aggression | YP are less angry, irritable, or aggressive, less prone to outburst, and better able to manager their temper. | “I would BREAK things, and I would like break my stuff that I really like […] but I don’t do that now at all, I just cry, that’s it.” (Jenny, 17 yrs, STPP) | | |
|  | Appetite | YP have a healthier appetite and weight. | “I started eating properly.” (Gemma, 16 yrs, BPI) | | |
|  | Sleeping and energy | YP have healthier sleep patterns and energy levels. | “working on the sleep was a bit of a quick win.” (Therapist of Connor, 18 yrs, CBT) | | |
|  | Self-harm | YP engage less in self-harm (e.g., cutting, trichotillomania). | “Well, certainly all the self-harm and thoughts of self-harm… cleared up within the first… 5, 6 weeks” (Therapist of Jenny, 17 yrs, STPP) | | |
|  | Suicidality | Reduced suicidal ideation and behaviour. | “First of all, I don’t feel suicidal anymore um and like to me I think that is the greatest improvement.” (Natalie, 15 yrs, STPP) | | |
|  | Anxiety | YP experience fewer fears, worries, panic attacks, or social anxiety; they engage in activities they previously avoided. | “I don’t feel as anxious like I haven’t had a panic attack since November or something.”(Poppy, 18 yrs, BPI) | | |
|  | Other comorbid issues | Improvements in less other comorbid problems such as substance use or obsessive-compulsive symptoms. | “The Class A’s and stuff that’s very rare, that’s sort of a party thing now.” (Dylan, 16 yrs, STPP) | | |
| Coping and self-management | Behavioural activation | YP become more active, return to hobbies or engage in new activities, including volunteering or work experience; YP gain a sense of purpose, routine and structure. | “The activity planning […] I think that’s the thing that helped the most […] just not sitting around doing nothing all the time but instead say actually being more sociable and... kind of getting back to... to the person I was before.” (Charlotte, 14 yrs, CBT). | | |
|  | Coping and resilience | YP learn specific coping strategies (e.g., breathing or counting exercises).  YP have a better understanding of their feelings and thoughts and how these link to behaviours; YP can anticipate and manage challenges.  YP are more resilient, with greater self-efficacy, and sense of control. | “If you're nervous before an exam she says take deep breaths, do the 10 seconds breath exercise that we did […] It actually helps.” (Madeline, 15 yrs, CBT)  “It did wake me up to how my o- sort of how it all works and like how my brain works and how to sort of react to certain things […] The fact that if you can understand something you can fix something, that’s my motto. So, if I can understand like in a computer game, if I can understand why it’s not working, I can fix the problem.” (Dylan, 16 yrs, STPP) | | |
|  | Cognition and behaviour | YP can challenge negative automatic thoughts, imagine alternatives, and approach situations differently, with more flexible thinking styles. | “I’m now able to think it through logically and think about different peoples’ point of view and stick it together and be more diplomatic about things and yeah just react to situations differently.” (Athena, 19 yrs, STPP) | | |
| Functioning | Global functioning | YP ‘function’ better across a range of life domains and are able to engage in activities considered typical for adolescence. | | “I guess I can sort of just do a bit more than I used to be able to… slightly more focused and erm just feeling a bit better as a whole.” (Stuart, 15 yrs, STPP) | |
|  | Executive functioning | YP are better able to get things done, due to improved concentration, motivation, planning and organisation. | | “I remember her giving me a sheet where, cause I have a very disorganised personality and during this time, school wise, I need that, I need that so my grades don’t fail completely. So, she tried to help me have a more organised personality. That didn’t really work, but it got me more thinking of how organised I should be.” (Adrian, 16 yrs, CBT) | |
|  | Academic and vocational functioning | YP attend school more regularly after frequent absence or a period of leave caused by the depression.  YP work more effectively in school and achieve better results. | | “If she’d have carried on being like she was last year at school there's no way she would get the grades that they are predicting.” (Mother of Mikayla, 15 yrs STPP)  “She’s staying in school, so she’s actually attending classes which she wasn’t, she was coming home too much, or sitting in the office not doing work, so she actually started doing lessons, she was paranoid about her GCSEs that she wouldn’t get any and she’s done the first year and she’s been predicted very high grades.” (Mother of Ella, 15 yrs, BPI) | |
|  | Social functioning | YP are more outgoing and talkative, more present within friendship groups and more socially connected.  YP find it easier to make conversation, relate to others, and be mindful of others’ feelings. | | “Linking up with friends, I mean this was something that we worked on quite a lot: Could she bear to actually link up with people that she may not know that well just for the sake of having somebody to go in the lunch queue with.” (Therapist of Jenny, 17 yrs, STPP) | |
| Personal growth | Assertiveness | YP are better able to stand up for their needs and opinions, overcome the urge to please, and can express disagreement or disapproval where appropriate. | | “I guess it’s almost thinking more about myself instead of trying to please other people and try to be like that, diplomatic to remember that my health is the most important thing.” (Athena, 19 yrs, STPP) | |
|  | Autonomy and responsibility | YP are more independent and able to take responsibility for their lives and actions. | | “When she speaks to her psychologist, maybe she gets advised that she is a grown young woman now and that she can make her own choices.” (Mother of Mikayla, 15 yrs, STPP) | |
|  | Identity | YP find out who they are and how to be themselves around other people; less idealised self-images that can accommodate both positive and challenging personality traits; positive and negative feelings. | | “I think that-that strengthened her enormously it actually gave her a much more rounded experience of herself... erm that she could acknowledge that she had erm... the bad and the good the sort of the kind parts and the... selfish parts of her and that-that they all went to make up (name of adolescent) the girl that she was.” (Therapist of Jenny, 17 yrs, STPP) | |
|  | Processing past and present | YP are able to make sense of challenging past or ongoing experiences such as bereavement, parental divorce, or family conflict. | | “I mean obviously, you know, the loss and the pain and all of that are still there but it became much more manageable for (name) and it wasn’t really-, I think the thing that had sort of troubled her the most was how alienated it had left her feeling and the conversations kind of healed that to an extent.” (Therapist of Louise, 17 yrs, CBT) | |
|  | Confidence and self-esteem | YP feel more confident within themselves, less insecure and vulnerable to the judgement of others, have higher self-regard. | | “I began feeling more confident about myself and I realise I'm not really as much of an idiot as I thought it was.” (Priya, 14 yrs, BPI) | |
| Feeling seen and seeing differently |  | YP felt listened to, understood, or cared for by another person, for the first time.  YP experienced being worthy of another person’s undivided attention.  YP felt therapy opened up new perspectives and ways of looking at things, or helped them see ’the bigger picture’.  YP had the opportunity to release feelings, thoughts or memories that had built up for a long time. | | | “I feel she unders-, understands me and she […] listens carefully to me you know. The things like that really help you know, just sometimes it’s nice just to be heard. (Poppy, 18 yrs, BPI)  “back then I felt like nobody cared about me and I don’t I think it made me feel good within myself because it was just it’s kinda what I needed like to feel like someone (breathes out) does care and that like they are there for me” (Natalie, 15 yrs, STPP) |
| Relationships | Ability to talk | YP feel more able to talk about feelings and thoughts, which helps deepen relationships; having a stronger support network also facilitates opening up. | | | “I think cause like I’ve talked about it s-so much that I’m still trying to find a way to um uh express myself correctly because before I had a problem I couldn’t e-express myself or tell people how I felt. But now I think like that’s something I’ve really improved on but I’m still improving on.” (Poppy, 18 yrs, BPI) |
|  | Family functioning and relationships | YP get on better with their family: less conflict as YP cope better and families understand them better; easing of entrenched tensions between family members; families communicate more openly; YP clarify their role within the family system. | | | “[Mother and daughter] come across like they get on much more and there is sort of better attunement between the two, a lighter, a lighter emotional quality to, to their relationship. Whereas before it was very sort of anxious, suspicious sort of just almost just not knowing each other.” (Therapist of Louise, 17 yrs, CBT) |
|  | Friendships | Reactivation or deepening of existing friendships, expanding friendship groups or changing friends by turning towards more supportive friendships. | | | “I think I’ve just changed the people that are around me and that’s helped a lot. I think erm…yeah, it’s a lot more grown-up people and more people that are a lot better at supporting me than previously I was friends with, which I think is very helpful.” (Athena, 19 yrs, STPP) |
|  | Peer relationships | Getting on better with peers in school (e.g., less bullying) or a romantic partner. | | | “I'm just living life really apart from the fact that I'm not bullied anymore.” (Priya, 14 yrs, BPI) |
| Wellbeing | Peace of mind | YP feel calmer, more balanced, relaxed, and carefree; YP fee as if a weight had been lifted off their shoulders; YP are more accepting of things they cannot change. | | | “She looked younger than 14 and it was almost like she was a little child carrying the world on her shoulders but as erm she seemed less burdened she was able to be in a way just be herself more.” (Therapist of Priya, 14 yrs, BPI) |
|  | Optimism | YP have a more positive and optimistic outlook into their lives and the future. | | | “I guess people probably notice that I’ve been a bit more optimistic about things.” (Adrian, 16 yrs, CBT) |
|  | Future orientation | YP can make plans for the future and have goals (e.g., applying for college or university, saving money to travel abroad) | | | “I made new friends started planning summer… yeah just getting excited for things, I dunno, just making plans I think was like… coz when you're really down you don’t make plans.” (Ada, 18 yrs, STPP) |

| Outcome domain | Outcome category | | Description | | | Example quote |
| --- | --- | --- | --- | --- | --- | --- |
| Parental support and wellbeing | | Parental support | | PAs are better able to understand their child’s difficulties and more aware of how their parenting practices may contribute to these difficulties.  Parents learn to support and parent their child more effectively. | “I think we’re a bit more self-aware. I'm not saying we don’t make the same mistakes I think we still make mistakes perhaps about what we do... but I think we’re more aware when we do it now... and then we try-we try and stop.” (Mother of Jenny, 17 yrs, STPP) | |
|  |  | Parental wellbeing | | PA feel less guilty, isolated, stressed, and worried; PA feel reassured, supported, and able to express their own frustrations and issues. | “As a mother… not having to be totally responsible for fixing and sorting something out… that is too big for you… urm so just... yes entering the system and... being able to relinquish some of that responsibility is positive in itself.” (Mother of Hayley, 16 yrs, STPP) | |

*Note.* PA = parents; CL = clinician; YP = young people; yrs = years.
